# Supplementary material for: Bone Growth and Development in Different Breeds of Piglets at the Early Age Is Associated with Mineral Deposition
Source: Animals (Basel). 2025 Dec 8;15(24):3536. doi: 10.3390/ani15243536 (PMC12729609; doi:10.3390/ani15243536)
Supplement: Supplementary file 1 [file animals-15-03536-s001.zip › animals-3971833-supplementary.pdf]

Supplementary Materials

Table S1. Ingredients and composition and nutrient levels of diets (dry-matter basis).

Item	Content, %
Ingredients	
Corn (8% CP)	22.70
Broken rice	8.50
Wheat flour	10.00
Soybean meal (46% CP)	5.50
Fermented soybean meal	6.00
Extruded soybeans (35.5% CP)	18.00
Pentapeptide	6.25
Fish meal (67% CP)	3.00
Fish soluble (55% CP)	1.25
Yeast hydrolysate (45% CP)	1.25
Fat powder	1.25
Limestone powder	0.30
$Ca(H_2PO_4)_2$	0.80
Whey powder (low-protein)	7.50
Glucose	6.25
Zinc oxide	0.20
L-Lysine hydrochloride (78.5%)	0.50
DL-Methionine (99%)	0.12
L-Threonine (98.5%)	0.13
Premix[1]	0.50
Total	100.00
Nutrient levels[2]	
Digestive energy, MJ/kg	14.20
Crude protein	18.11
Lysine	1.41
Methionine	0.45
Threonine	0.91
Calcium	0.65
Available phosphorus	0.45

[1] Per kilogram of premix containing 2,500 IU vitamin A, 575 IU vitamin D_3, 80 IU vitamin E, 0.8 mg vitamin B_1, 2 mg vitamin B_2, 7 mg vitamin B_6, 10 mg vitamin B_{12}, 10 mg niacin, 6 mg pantothenic acid, 2.5 mg biotin, 6 mg Cu ($CuSO_4$), 100 mg Fe ($Fe_2(SO_4)_3{\cdot}H_2O$), 100 mg Zn ($ZnSO_4{\cdot}H_2O$), 0.20 mg I ($Ca(IO_3)_2$), and 0.3 mg Se (Na_2SeO_3).

[2] Nutrient levels are measured values in triplicates.
